# Satisfaction with the Care Received and the Childbirth and Puerperium Experience in Christian and Muslim Pregnant Women

**DOI:** 10.3390/healthcare10040725

**Published:** 2022-04-13

**Authors:** Francisco Javier Fernández-Carrasco, Gustavo Adolfo Silva-Muñoz, Juana María Vázquez-Lara, Juan Gómez-Salgado, Juan Jesús García-Iglesias, Luciano Rodríguez-Díaz

**Affiliations:** 1 Department of Obstetrics, Punta de Europa Hospital, 11207 Algeciras, Spain; fjavier.fernandez@uca.es (F.J.F.-C.); guslavosm@hotmail.com (G.A.S.-M.); juana.vazquez@uca.es (J.M.V.-L.); 2 Nursing and Physiotherapy Department, Faculty of Nursing, University of Cádiz, 11207 Algeciras, Spain; 3 Department of Sociology, Social Work and Public Health, Faculty of Labour Sciences, University of Huelva, 21007 Huelva, Spain; juanjesus.garcia@dstso.uhu.es; 4 Safety and Health Postgraduate Programme, University of Espíritu Santo, Guayaquil 092301, Ecuador; 5 School of Health Sciences, University of Granada, 51003 Ceuta, Spain; lucianin000@gmail.com

**Keywords:** childbirth, childbirth experience, patient satisfaction, cultural diversity, Christianity, Islam, pregnant women, postpartum period

## Abstract

Over the last three decades, there has been an increase in the population as a result of the migratory flow due to the arrival of migrants to Spain, including young women of childbearing age and with reproductive capacity. This phenomenon has made childbirth assistance an extremely important priority in recent years. The aim of this study was to assess the satisfaction and experience during childbirth and the postpartum period in pregnant women according to their religion after assistance in a tertiary hospital. A descriptive cross-sectional study was conducted on a sample of 242 women using the validated Spanish version of the Mackey Childbirth Satisfaction Rating Scale (MCSRS) to measure satisfaction with the childbirth experience during the months of January to April 2021. Statistically significant differences were found in the domains of birth satisfaction (*p* < 0.01), satisfaction with the obstetrician (*p* < 0.01), and perception of pain during labour (*p* < 0.01). The Christian group of women scored higher in these three domains as compared to the Muslim group. The rate of breastfeeding at birth was 5.26 times higher among the Muslim group compared to the Christian group (*p* = 0.02). The experience of childbirth and the puerperium significantly influenced the levels of satisfaction of pregnant women with the process of childbirth in a different way according to the religious culture of the patient.

## 1. Introduction

Lived experiences during childbirth have the potential to impact women’s lives. The quality of these experiences is specifically related to fragility, vulnerability, stress, and emotional alterations that mark the experience and constitute memories of various kinds in pregnant women [1]. Throughout the pregnancy process, the woman creates expectations related to childbirth, generally established by cultural and social influences such as the support of her family network or access to and knowledge of the health system. If a good relationship is established between the health professional and the patient, the patient can gain positive reinforcement regarding her subsequent labour. If this link is not established, the woman is more likely to have doubts, conflicts or fears due to previous poor experience, lack of knowledge, and low confidence in the system [1,2].

The approach provided by Leininger’s nursing theory of diversity and universality of cultural nursing care [3] offers patients from different cultures the opportunity to express their wishes by guiding professionals to achieve culturally appropriate health care. Social life cannot be conceived without a religious dimension, since it is through it that the moral status that governs a society can be analysed, as well as the worldview around it, which gives a unique identity to the residing inhabitants. Culture and religion are not two distinct spheres of social life but part of a general construct that defines the creation of societies. This is why the study of their interrelationship helps us understand the dimensions of culture more precisely, insofar as religion is a human creation and activity that is conceivable only and thanks to society itself. Religion gains strength as a cultural phenomenon because it helps build personality in childhood and ensure social cohesion [4]. In this research, when referring to both Christian and Muslim culture, we do not refer to religion as person’s relationship with a divinity but rather as the set of beliefs, behavioural norms, and morality that are associated with culture.

Likewise, over the last three decades, there has been an increase in the population as a result of the flow of migrants to Spain, including young women of childbearing age and with reproductive capacity. This phenomenon has made childbirth assistance an extremely important priority in recent years [5]. In this sense, the different cultural approaches that people from other cultures may take to pregnancy, childbirth, and puerperium depending on their country of origin must be considered, and this factor could have a significant influence on the perception and experience of childbirth [6]. Cultural aspects are fundamental factors in the perception and acceptance of health services by migrant women, as the literature shows that their reproductive behaviours are influenced by the socio-cultural and health conceptions of their country of origin [7,8]. According to the Spanish Statistical Office, immigrants living in Spain correspond to 11.5% of the population. In terms of religion, 97% of the Spanish population is Christian, 2% is Muslim, and the remaining 0.7% belongs to other minority religions [9].

In the field of obstetrics, maternal satisfaction after childbirth has consequences for the health of the mother and her newborn [10], so its measurement could provide a valuable result to improve the quality of care provided [11]. Some authors relate satisfaction to the fulfilment of women’s expectations of care [10]. In this sense, Hodnett identifies the personal expectations of pregnant women, women’s participation in decision-making, and the support and quality of the relationship with health professionals [11] as key elements for experience satisfaction. Additionally, a positive birth experience may have implications in the satisfaction of a subsequent pregnancy, as it has been observed that, in these cases, women are more likely to have positive expectations for their next birth [12].

The concept of satisfaction with the experience of childbirth and with the health care received during this process is currently an important indicator of the quality of maternity care [13]. Despite this, it is difficult to measure factors such as perceived quality and satisfaction with health care, since, as previously mentioned, women construct their experience of childbirth differently depending on multiple emotional, social, cultural, and psychological factors [14]. Self-esteem and autonomy can be affected by the support a mother receives, how she prepares for childbirth, and how she accepts her feelings before, during, and after birth [13]. Having a positive experience of motherhood can improve interaction with the child, which may lead to a strong foundation for long-term family health. However, satisfaction is a life-changing event, and positive birth satisfaction outcomes are subject to a variety of individual and environmental factors that must be considered in any assessment [13].

Therefore, the aim of this study was to assess the satisfaction and experience during childbirth and the postpartum period in pregnant women after being assisted in a tertiary hospital according to whether they are socialised in Christian or Muslim culture.

## 2. Materials and Methods

### 2.1. Design and Participants

A descriptive cross-sectional study was designed. The sample consisted of women who gave birth to full-term children at the Hospital Punta de Europa in Algeciras (Spain) from 1 January 2021 to 30 April 2021. This hospital is considered a Level 3 hospital. The Sample Size Calculator was used to calculate the sample size. A confidence level of 95% with a margin of error of 0.05 was established. The total population was estimated at 366, and for these data, the sample size should be at least 188 individuals. Eventually, a sample of *n* = 242 was obtained. The sample was obtained from a consecutive sampling of all women visiting the centre to give birth who met the inclusion criteria and wished to participate in the study on a voluntary basis. The two groups were configured as follows: 176 subjects represented the Christian culture group and 66 subjects represented the Muslim culture group.

The inclusion criteria were as follows: (1) women for whom the birth was normal, without any complications beyond the usual ones, be it normal birth, instrumental, or caesarean section; (2) women whose newborns were born healthy, without any complications and not requiring more time in hospital than usual; (3) women who were Christian or Muslim; and (4) women who were fluent in Spanish, both spoken and written.

In the study period, a total of 360 births were attended in the study hospital. Of these patients, 261 were Christian, 98 were Muslim, and only 1 declared a different religion. In total, there were 11 Muslim women who did not speak or understand Spanish. In terms of maternal complications during childbirth, there was only one case of severe postpartum haemorrhage, and in terms of neonatal complications, there were only 2 cases in which the newborns required more time in hospital than usual.

After informed consent was requested, the patient was asked to fill in the questionnaire before her discharge from hospital. Data collection was carried out by the research team. 

### 2.2. Measuring Instrument

A self-administered questionnaire consisting of one part including socio-demographic data and a second part focused on a Spanish validated questionnaire of the Mackey Childbirth Satisfaction Rating Scale (MCSRS) [13] was used as the measuring instrument. The MCSRS was originally developed in English, and it was administered to the woman after delivery. In 2012, Mas-Pons et al. [14] validated the scale in Spanish to measure the satisfaction after the experience of childbirth, obtaining a Cronbach’s alpha coefficient of 0.94 for the total scale. The scale consists of 34 items grouped into five domains referring to the woman (9 items), the partner (2 items), the newborn (3 items), the midwife (9 items), and the obstetrician (8 items). It also contains an overall experience rating subscale (3 items). Each item is evaluated on a 5-point Likert scale ranging from very dissatisfied (1) to very satisfied (5). The final score of the scale is obtained by adding the values assigned to each item, so the higher the score, the higher the satisfaction. In addition, the scale allows partial scores to be given for each subscale.

### 2.3. Statistical Analysis

Descriptive statistics were presented as percentages and frequencies. The Kolmogorov–Smirnov test was used to determine whether the data showed normal behaviour; the Kruskal–Wallis test was used to test whether there were differences in the analysed groups with respect to the assessment of the different dimensions; and the Mann–Whitney U test, with Bonferroni correction, was used to analyse which subgroups differed from each other.

Finally, a binary logistic regression analysis was performed on those factors that were considered most influential after the preliminary study (marital status, level of education, income, presence of a companion at birth, and breastfeeding after the first hour). To study the goodness of fit of the logistic regression model, the Nagelkerke R-squared test and the Hosmer and Lemeshow test were used.

SPSS software version 20.0 was used for the study, and a confidence level equal to or greater than 0.05 was established for the entire analysis as a criterion for considering that a statistically significant effect exists. 

### 2.4. Ethical Considerations

Participants answered the questionnaire voluntarily and accepted the informed consent. Participants’ responses were recorded anonymously and the information was treated confidentially in accordance with Organic Law 3/2018, of 5 December, on Personal Data Protection and guarantee of digital rights.

In addition, this study was approved by the Research Ethics Committee of Cadiz as a body attached to the Ministry of Health and Families of the Andalusian Regional Government (Spain) in April 2021.

## 3. Results

Table 1 presents the sociodemographic characteristics of the sample. A total of 242 patients were included, of whom 72.7% belonged to the Christian culture group (*n* = 176) and 27.3% belonged to the Muslim culture group (*n* = 66).

Table 2 shows the means of the different test domains as well as the total score.

According to Table 3, women of Christian culture obtained a higher mean score in the pain (M = 8.31, SD = 1.79), dilation phase (M = 21.05, SD = 5.04), expulsive phase (M = 18.63, SD = 4.55), and obstetrician (M = 27.09; SD = 6.05) domains; on the other hand, women of Muslim culture obtained a higher mean score in the midwife (M = 28.03; SD = 3.31) and newborn (M = 10.30; SD = 1.85) domains. Statistically significant differences were found in the pain, satisfaction with the delivery, and satisfaction with the obstetrician variables. In terms of overall satisfaction with the questionnaire, the Christian group (M = 6.96, SD = 1.44) scored slightly higher than the Muslim group (M = 6.82, SD = 1.20), but these differences were not statistically significant (*p* = 0.11).

A binary logistic regression model was used for the multivariate analysis. Culture was taken as the dependent variable, and all variables that were statistically significant in the bivariate analysis were taken as independent variables. These variables were marital status, level of education, income, presence of a birth companion, initiation of breastfeeding after the first hour of labour, pain experienced during labour, methods of pain relief, satisfaction during the second stage of labour, and satisfaction with the obstetrician (Table 4).

According to this model, the following results were found as regards marital status:

Muslim women were 4.16 times more likely to be married than single, compared to Christian women.

With regard to educational level, Christian women were 1.92 times more likely to have university education than no studies, compared to Muslim women.

In terms of income, Christian women were 14.19 times more likely to have an income of more than EUR 2000 than no income, compared to Muslim women.

In relation to accompaniment during birth, the model indicates that Christian women were 17.9 times more likely to be accompanied than being alone, compared to Muslim women.

Finally, the model predicts that, in relation to the initiation of breastfeeding after the first hour postpartum, Muslim women were 5.26 times more likely to breastfeed their newborns than Christian women.

## 4. Discussion

The main findings of this study were that statistically significant differences were found in the domains of birth satisfaction, satisfaction with the obstetrician, and perception of pain during labour. The Christian group of women scored higher in these three domains compared to the Muslim group. The rate of breastfeeding at birth was 5.26 times higher among the Muslim group compared to the Christian group.

There are few studies in the literature that measure the satisfaction with the birth experience [11,12,13,14,15]. In this case, the experience was measured in women from different cultures, specifically Muslim women from the north of Morocco and Christian women from Spain.

Studies in the UK, Australia, Canada, and Sweden have found that continuous support from health care staff, a close relationship with them, and a warm atmosphere in birth centres may be factors that encourage women to obtain more information, participate in decision making, and have greater overall satisfaction [16,17].

Handelzalts et al. [18] conducted a study to assess the experience of childbirth in emergency situations in the second stage of labour. They concluded that, in instrumental deliveries and emergency caesarean sections, women were less satisfied than those who did not undergo an emergency intervention. In the present study, it was Christian women who had a higher number of caesarean sections and instrumental deliveries compared to Muslim women, and the latter had a higher level of satisfaction, although with no statistically significant differences. A recent study [19] compared the level of satisfaction with the birth experience when accompanied or unaccompanied. This study indicated that women who were accompanied during labour had higher levels of satisfaction with the birth experience than those who were not. In this study sample, the proportion of Christian women who were accompanied at birth was much higher than in the Muslim group. An odds ratio of 5.01 was identified; i.e., Christian women were five times more likely to be accompanied at birth than Muslim women. Although there was no significant difference, these data agree with the previous study that Christian women scored higher levels of satisfaction than Muslim women.

Many studies [18,19,20,21,22] have shown that the response to pain varies according to culture. As for the perception of the pain of labour contractions, both the perception and the expression of pain may be socio-culturally constructed [23,24]. In some cultures, women in labour pain are urged to remain silent, while in other cultures they are allowed to express their pain [25]. Although not generalisable to all women, those from North Africa tend to be less inhibited and more exaggerated in their expression of pain. Many Muslim women are reluctant to use epidural analgesia, often due to lack of knowledge [26]. In the present study, the percentage of Muslim women who received epidural analgesia was 33%. However, in women of Christian culture, about 60% made use of the different methods of pain relief. The perception of pain may be influenced by other issues such as pre-birth information, e.g., attendance of childbirth preparation classes, as well as different psychosocial conditions that women may have; e.g., increased fear of the birth process, loneliness, and lack of emotional support predisposes women to a lower pain tolerance [25]. Eighty-five per cent of the sample did not attend childbirth preparation classes, which could have caused a higher perception of pain, but there is little difference between women from Christian and Muslim cultures in this respect, as the proportion between the two groups was similar. In a study on Islamic women [26], it was shown that those who received pre-birth information had significantly reduced levels of pain during labour. Although Luque-Fernández and Oliver-Reche [25] stated that Muslim women are more vocal when it comes to expressing their pain in labour, in our case the opposite was true, with a greater verbalisation and expression of feelings about the perception of pain being higher for the group of Christian women than for the group of Muslim women, and statistically significant differences were found in this respect. Another recent study correlated accompaniment during the birth process with perceived pain during labour. The authors indicated that patients who were accompanied at birth reported less pain than those who were not accompanied [27].

On the other hand, the World Health Organisation recommends exclusive breastfeeding until the sixth month of life and mixed breastfeeding until 2 years or more. However, at least 85% of mothers in the world do not follow these recommendations, and only 35% of infants under 4 months of age are exclusively breastfed [28]. In the present study, Christian women breastfed less than Muslim women in the first hour of life. Muslim women were 5.26 times more likely to breastfeed their baby in the first hour of life than Christian women.

Regarding the possible limitations, the first difficulty encountered is the small number of studies available in the scientific literature that assess levels of satisfaction with the birth experience, and it is even more difficult to find those which relate the birth experience to other cultures. Secondly, another limitation may be that the proportion of women of Muslim culture is smaller than the proportion of women of Christian culture, so comparisons may be affected even though the sample was significant. In addition, the type of sampling used, i.e., non-probabilistic, allows for an orientation of the results, but not a representativeness of the sample. In this sense, it should be noted that the results point to associations, but do not allow us to establish cause-and-effect relationships, as it is a cross-sectional study. Finally, another possible limitation may lie in the fact that the sample was drawn from a single hospital. The results obtained should not be generalised to other different populations; however, due to the proximity to North Africa, the population may be representative of both cultures. Further research would be necessary to assess satisfaction with the birth experience in order to adapt and improve the health services offered to the population regardless of their culture.

## 5. Conclusions

Satisfaction in childbirth can be influenced by the woman’s culture, as women of a Christian culture show greater satisfaction in general. Nevertheless, they perceive the birth experience as more painful given the use of epidural analgesia. In addition, they have more dystocic deliveries, leading to higher satisfaction with the obstetrician.

On the other hand, women of Muslim culture have a higher number of eutocic deliveries and score higher in terms of satisfaction with the midwife. They also show higher rates of breastfeeding and were less accompanied by a family member during the dilation and expulsive phases of labour.

## Figures and Tables

**Table 1:** Description of sociodemographic characteristics.

Quantitative Variables						
Variable	Minimum	Maximum	M	SD ^1^		N
Age	19	44	30.59	5.38		242
Number of children	0	5	1.69	0.89		242
**Qualitative Variables**						
				**N**		**%**
Marital status	Single			104	103 Christians	43%
					1 Muslim	
	Married			130	65 Christians	53.7%
					65 Muslims	
	Separate/Divorced			6	6 Christians	2.5%
					0 Muslim	
	Widow			2	2 Christians	0.8%
					0 Muslims	
Educational level	No studies			15	5 Christians	6%
					10 Muslims	
	Primary education			70	40 Christians	29%
					30 Muslims	
	High School/Vocational			97	80 Christians	40%
					17 Muslims	
	University/Post-Graduate			60	51 Christians	25%
					9 Muslims	
Culture	Christian			176		72.7%
	Muslim			66		27.3%

^1^ SD: Standard Deviation.

**Table 2:** Test domains and total score.

Variables	N	Minimum	Maximum	M	SD ^1^
Pain domain	242	2	10	8.13	1.87
Dilatation domain	242	4	28	20.99	4.88
Expulsive domain	242	6	24	18.31	4.44
Newborn domain	242	4	12	10.29	1.97
Midwife domain	242	6	32	27.64	5.05
Obstetrician domain	242	4	32	26.78	5.82
Overall experience domain	242	0	8	6.92	1.37
Final score	242	37	136	110.93	19.29

^1^ SD: Standard Deviation.

**Table 3:** Comparison between means according to the culture.

Domains	Culture			*p*-Value ^1^
Pain	Christian	M	8.31	0.01 *
		SD	1.79	
		N	176	
	Muslim	M	7.64	
		SD	2.03	
		N	66	
Dilatation	Christian	M	21.05	0.69
		SD	5.04	
		N	176	
	Muslim	M	20.85	
		SD	4.46	
		N	66	
Expulsive	Christian	M	18.63	0.01 *
		SD	4.55	
		N	176	
	Muslim	M	17.48	
		SD	4.05	
		N	66	
Newborn	Christian	M	10.28	0.7
		SD	2.03	
		N	176	
	Muslim	M	10.30	
		SD	1.85	
		N	66	
Midwife	Christian	M	27.49	0.46
		SD	5.57	
		N	176	
	Muslim	M	28.03	
		SD	3.31	
		N	66	
Obstetrician	Christian	M	27.09	0.01 *
		SD	6.05	
		N	176	
	Muslim	M	25.94	
		SD	5.09	
		N	66	
Overall experience	Christian	M	6.96	0.11
		SD	1.44	
		N	176	
	Muslim	M	6.82	
		SD	1.20	
		N	66	

^1^ U de Mann–Whitney; * *p* < 0.05.

**Table 4:** Binary logistic regression model for culture.

	Exp (B)	95% C.I. to EXP (B) ^1^		Sig.
		Lower	Higher	
**Marital status**				
Single	Ref.			0.003
Married	0.24	0.06	0.87	0.03
Separate/Divorced	1.34	0.39	4.53	0.63
Widow	0.11	0.008	1.62	0.10
**Educational level**				
No studies	Ref.			0.006
Primary education	5.74	0.40	81.55	0.19
High School/Vocational	0.27	0.07	1.01	0.05
University/Post-Graduate	1.92	0.66	5.55	0.22
**Incomes**				
None	Ref			0.005
EUR <500	26.81	2.84	252.6	0.004
EUR 500–1000	3.13	0.20	48.28	0.41
EUR 1000–2000	13.46	2.85	63.58	0.001
EUR >2000	14.19	3.16	63.64	0.001
Presence of a birth companion	17.90	6.56	48.78	0.01
Breastfeeding after the first hour postpartum	0.19	0.06	0.54	0.002
Constant	0.02			0.01

^1^ Hosmer–Lemeshow test (Chi squared = 11.71) *p* = 0.16 and Nagelkerke’s R^2^ (0.51).

## Data Availability

All data are available within this article.
